# The association between continuity of care and surgery in lumbar disc herniation patients

**DOI:** 10.1038/s41598-021-85064-1

**Published:** 2021-03-10

**Authors:** Eun-San Kim, Chang-yup Kim

**Affiliations:** Graduate School of Public Health, Seoul National University, 1 Gwanak-ro, Gwanak-gu, Seoul, 08826 Republic of Korea

**Keywords:** Pain, Health policy, Public health, Epidemiology

## Abstract

Continuity of care is a core dimension of high-quality care in the management of disease. The purpose of this study was to investigate the association between continuity of care and lumbar surgery in patients with moderate disc herniation. The Korean National Sample Cohort was used. The target population consisted of patients who have had disc herniation more than 6 months and didn’t get surgery and red flag signs within 6 months from onset. The population was enrolled from 2004 to 2013. The Bice-Boxerman Continuity of Care was used in measuring continuity of care. The marginal structural model with time dependent survival analysis was used. In total, 29,061 patients were enrolled in the cohort. High level of continuity of care was associated with a lower risk of lumbar surgery (HR, 0.27; 95% CI, 0.20–0.27). When the index was calculated only with outpatient visits to primary care with related specialty, the HR was 0.49 (95% CI: 0.43–0.57). In exploratory analysis, patients with lumbar stenosis and spondylolisthesis had higher risk of having a low level of continuity of care. These results indicate that continuity of care is associated with lower rates of lumbar surgery in patients with moderate disc herniation.

## Introduction

In the treatment of lumbar disc herniation, surgery is recommended for patients with severe symptoms^1^. Though surgery can alleviate the pain and improve physical function in a short-term, its long-term effect remains controversial^2–4^. Furthermore complications, such as recurrent lumbar disc herniation might occur^5^. Thus, in the management of patients with moderate or less severe disc herniation, the prevention of worsening of symptoms, which can lead to lumbar surgery, seems to be important.

The authors focused on the continuity of care to prevent surgery. Continuity of care is a core dimension of primary care^6^. It is defined as coherent, connected, and consistent care within patient's medical needs and personal context^7^, which is the opposite of discrete, separated, and uncoordinated care. It includes several aspects of high quality of care, such as organized collection of patient's information, patients receiving most of their health care, and interpersonal relationship between patient and health care provider^8^. Continuity of care is associated with patient's satisfaction^9^, quality of life^10^, medical costs^11^, and prevention of avoidable hospitalization^12^.

Continuity of care changes over the period of patient's disease episode. Walraven et al. pointed out the needs to consider continuity of care as time-varying exposure to avoid time-dependent bias^13^. Some previous studies use time-discrete designs, such as time-dependent survival analysis^14,15^ or generalized estimating Eq. ^16^. However, in epidemiology, it was pointed out that bias could be introduced in time-varying confounders. In the study design of continuity of care, time varying confounders are associated with previous and subsequent continuity of care. To solve this issue, a marginal structural model was suggested, which could control time-varying bias using inverse probability of treatment weighting (IPTW)^17^. The objective of this study was to investigate the association between continuity of care and surgery in patients with lumbar disc herniation using the marginal structural model.

## Results

### Baseline characteristics

**Figure 1:**
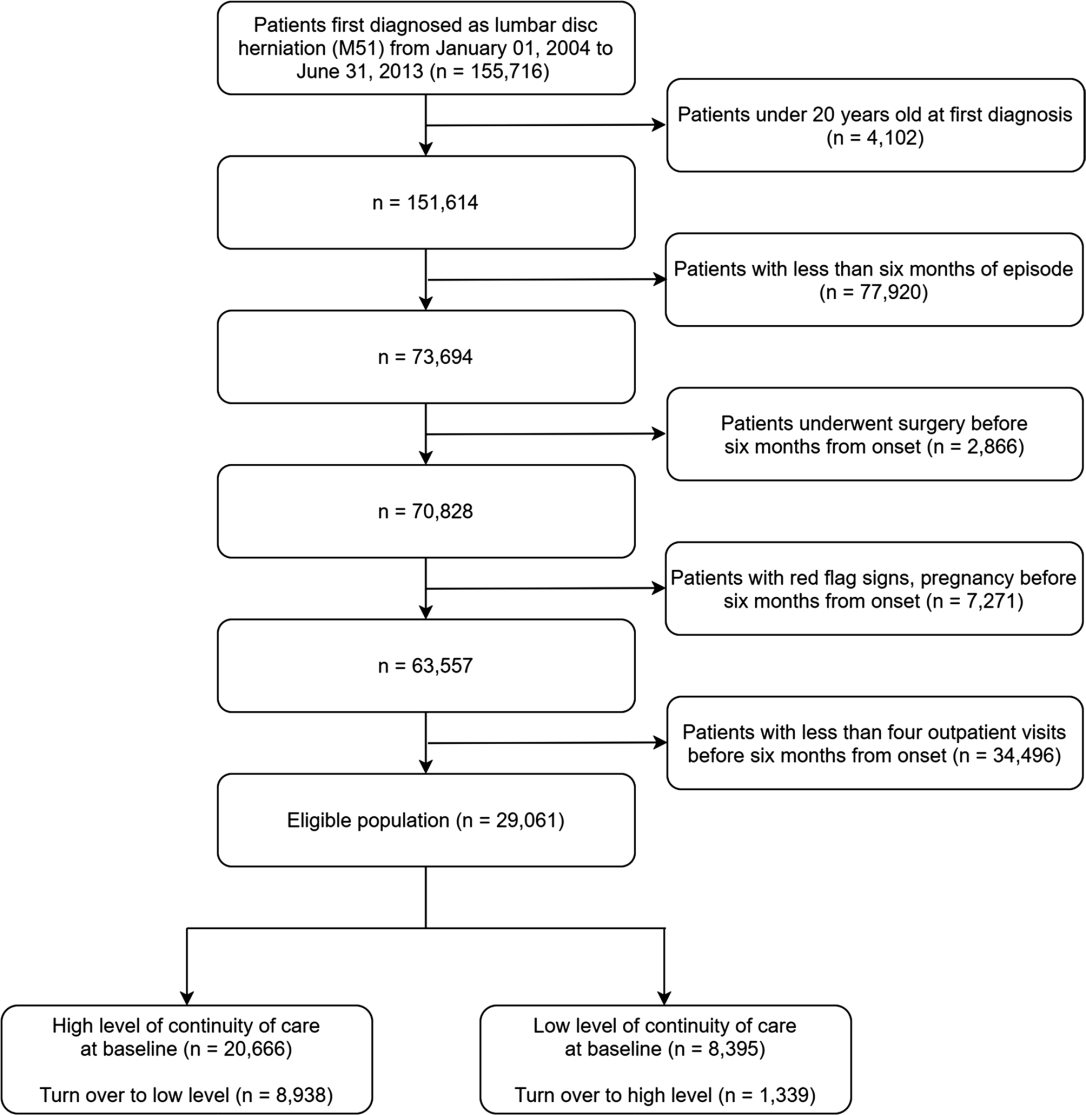
The flowchart of study population.

Among the 29,061 eligible cohort, 20,666 (71.1%) patients started with high level of continuity of care and 8395 (28.9%) patients started with low level of continuity of care (Fig. 1). At baseline, the proportion of females, the mean age was higher in patients having high level of continuity of care. The prevalence of lumbar stenosis and spondylolisthesis was higher in the patients having low level of continuity of care. The mean of number of outpatient visits and the utilization of medical interventions except antipsychotics was higher in the patients having low level of continuity of care (Table 1).

**Table 1:** Baseline characteristics of the study population.

	Continuity of care	
	High level (n = 20,666)	Low level (n = 8395)
Continuity of care index	1.00 ± 0.00	0.58 ± 0.19
	1.00 [1.00, 1.00]	0.56 [0.46, 0.73]
**Sex**		
Female	12955 (62.7)	4669 (55.6)
Male	7711 (37.3)	3726 (44.4)
**Age**	54.33 ± 14.76	49.74 ± 14.48
20–29	1156 (5.6)	795 (9.5)
30–39	2536 (12.3)	1430 (17.0)
40–49	4016 (19.4)	1905 (22.7)
50–59	4927 (23.8)	2014 (24.0)
60-	8031 (38.9)	2251 (26.8)
**Residence**		
Metropolitan	9075 (43.9)	3764 (44.8)
Urban	2551 (12.3)	944 (11.2)
Rural	9040 (43.7)	3687 (43.9)
**Income**		
High	7453 (36.1)	3000 (35.7)
Middle	5930 (28.7)	2282 (27.2)
Low	7283 (35.2)	3113 (37.1)
**Current working**	7423 (35.9)	3043 (36.2)
**Cohort entry**		
2004	1569 (7.6)	510 (6.1)
2005	2777 (13.4)	1020 (12.2)
2006	2867 (13.9)	1054 (12.6)
2007	2444 (11.8)	1046 (12.5)
2008	2085 (10.1)	839 (10.0)
2009	1938 (9.4)	757 (9.0)
2010	1977 (9.6)	870 (10.4)
2011	1987 (9.6)	833 (9.9)
2012	1658 (8.0)	805 (9.6)
2013	1364 (6.6)	661 (7.9)
**Physical disability or brain lesion disorder**	888 (4.3)	291 (3.5)
**Charlson comorbidity index**	1.57 ± 1.70	1.48 ± 1.63
**Osteoarthritis **	5667 (27.4)	1923 (22.9)
**Rheumatoid arthritis **	3404 (16.5)	1312 (15.6)
**Osteoporosis**	11834 (57.3)	4424 (52.7)
**Lumbar stenosis**	5078 (24.6)	2849 (33.9)
**Spondylolisthesis**	1167 (5.6)	580 (6.9)
**The number of outpatient visits**	11.92 ± 12.55	15.47 ± 14.83
**Hospitalization**	841 (4.1)	1167 (13.9)
**Non-steroidal anti-inflammatory drugs**	16643 (80.5)	7635 (90.9)
Cumulative number of prescribed days	20.30 ± 33.87	28.03 ± 32.12
**Steroids**	5365 (26.0)	3570 (42.5)
Cumulative number of prescribed days	1.68 ± 7.89	2.32 ± 7.00
**Opioids**	7725 (37.4)	4203 (50.1)
Cumulative number of prescribed days	5.00 ± 16.76	6.90 ± 16.66
**Anticonvulsants**	1134 (5.5)	849 (10.1)
Cumulative number of prescribed days	1.63 ± 12.67	2.30 ± 11.54
**Antidepressants**	2183 (10.6)	1190 (14.2)
Cumulative number of prescribed days	8.16 ± 40.52	8.16 ± 38.04
**Antipsychotics**	281 (1.4)	107 (1.3)
Cumulative number of prescribed days	1.53 ± 21.05	1.12 ± 15.10
**Anxiolytics**	8476 (41.0)	3791 (45.2)
Cumulative number of prescribed days	21.87 ± 57.02	21.12 ± 53.38
**Hypnotics**	1666 (8.1)	835 (9.9)
Cumulative number of prescribed days	2.43 ± 18.75	2.41 ± 18.94
**Epidural steroid injection**	3579 (17.3)	2838 (33.8)
Cumulative number of usages	0.61 ± 1.88	1.04 ± 2.27

The characteristics of population were presented according to the levels of continuity of care at baseline (6 months from onset). The median of continuity of care index is used for differentiating high and low levels of continuity of care. Continuous variable is presented with mean ± standard deviation. In continuity of care index, median [1st quartile, 3rd quartile] is also presented. Categorical variable is presented with number (percentage). All medical usages from onset to baseline was used for covariates, except that the number of outpatient visits and hospitalization were considered as time-varying confounder.

### The changes in continuity of care and hospitalization

**Figure 2:**
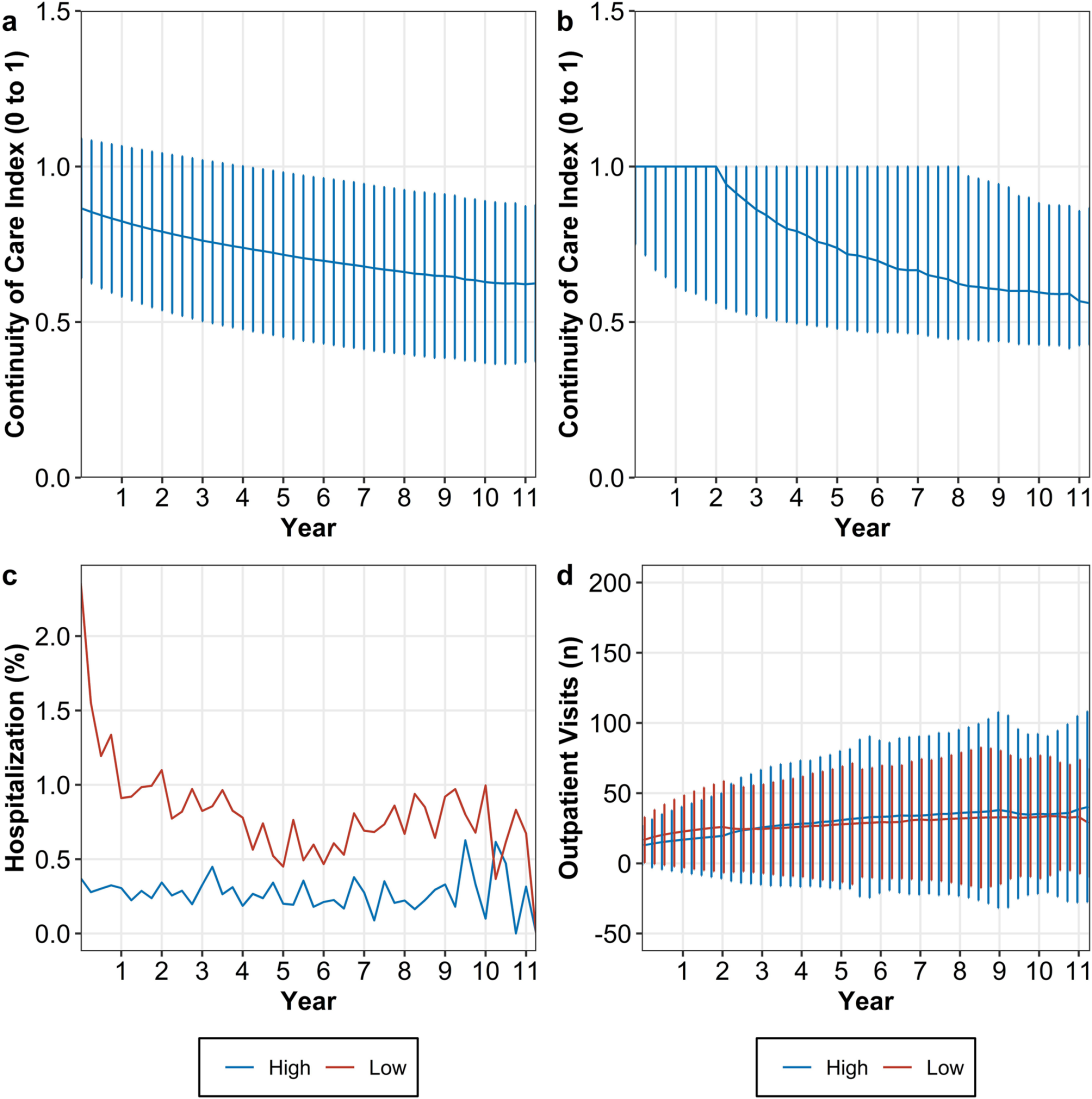
Time trend of continuity of care index and time-varying confounders. Time trend of continuity of care index is presented with (**a**) mean ± standard deviation (SD); (**b**) median, 25 and 75th quartiles. The events of hospitalization is presented with (**c**) proportion of hospitalized patients by the continuity of care level. The cumulative number of outpatient visits is presented with (**d**) mean ± SD. All values are presented by 3 months interval. The median of continuity of care by 3 months interval is used for differentiating high and low levels of continuity of care.

The longest follow-up time was 11.5 years and changes in the total number of patients over the follow up periods are presented in Supplementary Table S1. The median of continuity of care index was 1.00 [1st quartile: 0.75; 3rd quartile: 1.00] when follow-up begun and it declined to 0.56 [1st quartile: 0.43; 3rd quartile: 0.87] at the end of the cohort. Patients having low level of continuity of care were more likely to be hospitalized during periods. The mean of cumulative number of outpatient visits was higher in the patients with low levels of continuity of care until 3 years passed and the trend became reversed (Fig. 2).

### IPTW distribution

The distribution of IPTW is presented in Supplementary Fig. S1 and Supplementary Table S2. The median of IPTW was 0.993, 1st quartile was 0.979 and 3rd quartile was 1.001.

### The effects of continuity of care on lumbar surgery

There were 1,216 cases of lumbar surgery in this study. The high level of continuity of care was associated with a decreased risk of lumbar surgery in marginal structural model (HR, 0.23; 95% CI, 0.20–0.27). The HR of unweighted model was consistent with marginal structural model (HR, 0.24; 95% CI, 0.20–0.27). (Table 2).

**Table 2:** The association between continuity of care and lumbar surgery in patients with lumbar disc herniation.

Unadjusted	Multivariate adjusted model	Marginal structural model
0.20 (0.18–0.23)	0.24 (0.20–0.27)	0.23 (0.20–0.27)

The main analysis is marginal structural model. Marginal structural model was weighted by IPTW. With IPTW weighting, baseline confounders were adjusted. When baseline confounders did not meet PH assumption, the interaction term with time was included. In multivariate adjusted model, all confounders including time-varying confounders were adjusted in the model with no weights adjustment. The hazard ratio is presented with 95% confidence interval.

### Sensitivity analysis

Results of the sensitivity analysis are in Table 3. The result was still significant with 0.5 criteria (HR: 0.35; 95% CI: 0.31–0.39). When the index was calculated only with outpatient visits to primary care, the HR generally increased and with related specialty, the HR was 0.50 (95% CI: 0.44–0.58). The HR increased with patients who visited hospital within 6 months from onset but the results was significant (HR: 0.34; 95% CI: 0.28–0.42). The trend was consistent with patients enrolled after 2010 (HR: 0.22; 95% CI: 0.17–0.29).

**Table 3:** The results of sensitivity analysis.

Analysis set	Type of continuity of care index	Unadjusted	Multivariate adjusted model	Marginal structural model
Main set	Total outpatient visits with 0.5 criteria	0.30 (0.27–0.34)	0.35 (0.31–0.39)	0.35 (0.31–0.39)
	Primary care	0.44 (0.39–0.51)	0.51 (0.44–0.58)	0.50 (0.44–0.58)
	Primary care with related specialties	0.44 (0.39–0.51)	0.50 (0.44–0.58)	0.50 (0.44–0.58)
Patients who visited hospital within 6 months	Total outpatient visits	0.33 (0.28–0.41)	0.34 (0.28–0.42)	0.34 (0.28–0.42)
	Primary care	0.57 (0.43–0.75)	0.61 (0.46–0.81)	0.61 (0.46–0.81)
	Primary care with related specialties	0.57 (0.43–0.75)	0.61 (0.46–0.81)	0.60 (0.46–0.80)
Patients enrolled after 2010	Total outpatient visits	0.19 (0.15–0.25)	0.22 (0.17–0.29)	0.22 (0.17–0.29)
	Primary care	0.44 (0.33–0.58)	0.51 (0.38–0.68)	0.50 (0.38–0.66)
	Primary care with related specialties	0.44 (0.33–0.58)	0.50 (0.38–0.68)	0.50 (0.37–0.66)

Sensitivity analysis was performed according to criteria for differentiating level, index calculation and other analysis set. First, the high and low level of continuity of care was categorized by 0.5. Second, the index was calculated only by the outpatient visits to primary care. Third, the COC was calculated only by the outpatient visits to primary care with related specialties of the treatment of HDD. For other analysis set, first, patients who visited the hospital more than once from onset to baseline were analyzed. Second, patients registered in cohort after 2010 were analyzed. The hazard ratio is presented with 95% confidence interval.

### Subgroup analysis

**Figure 3:**
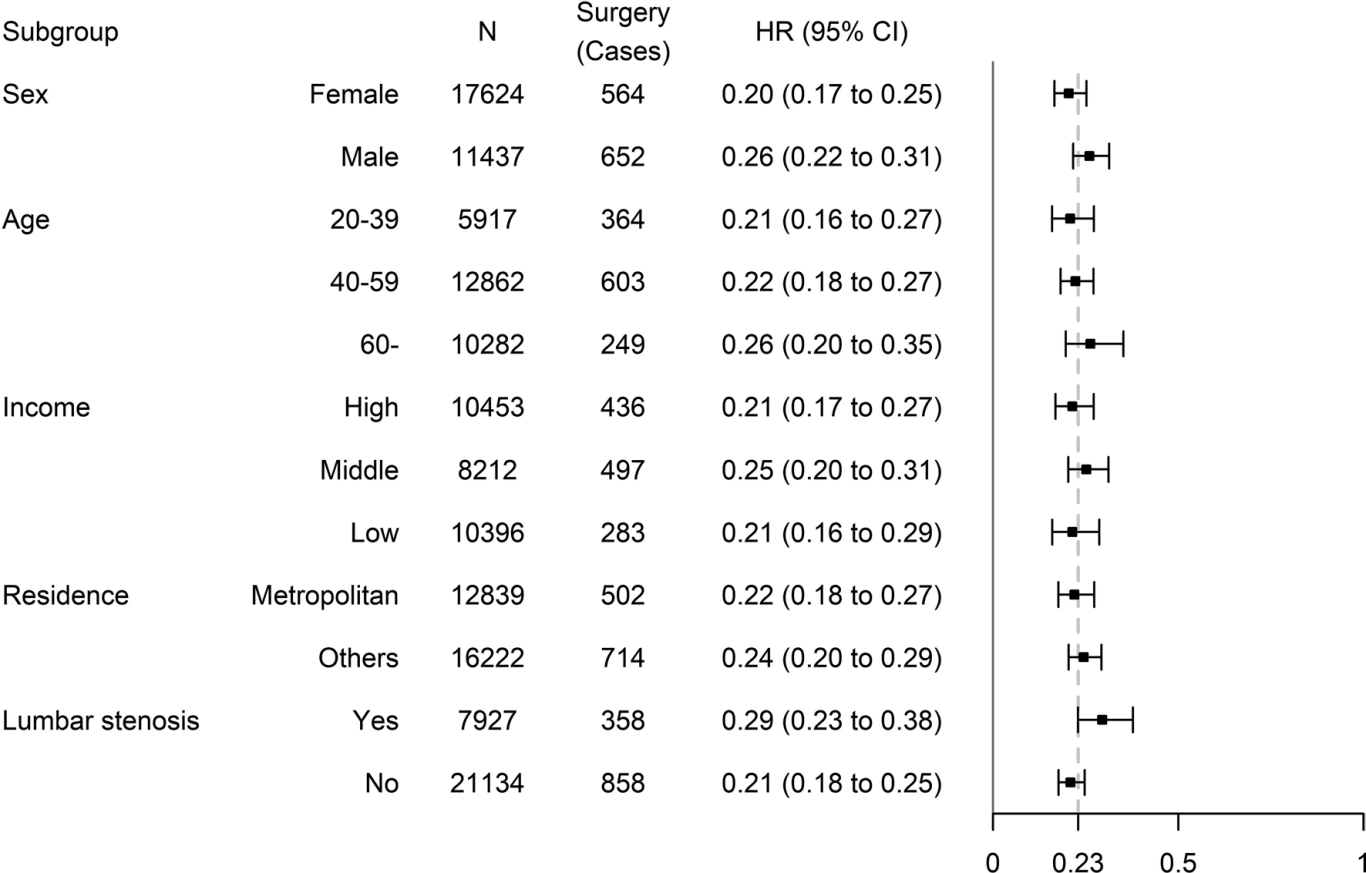
The subgroup analysis of the effects of continuity of care on lumbar surgery in patients with moderate lumbar disc herniation. The main analysis is marginal structural model. The dot line is the hazard ratio (HR) of the main analysis (HR: 0.23). The HR is presented with 95% confidence interval.

The trend was consistent in all subgroups. However, the HR slightly increased in the older group (More than 60 years old, HR: 0.26; 95% CI: 0.20–0.35). The HR was highest in the group with lumbar stenosis (HR: 0.29; 95% CI: 0.23–0.38) (Fig. 3).

### Exploratory analysis on turnover of continuity of care level

The results of the exploratory analysis are presented in Supplementary Fig. S2 and Supplementary Table S3. There were more events of change to other levels of continuity of care in patients having high levels of continuity of care at baseline (High: 8,938 (43.2%); Low: 1,339 (15.9%)), and the HR was 3.74 (95% CI: 3.32–3.74).

In patients having high level of continuity of care at baseline, older patients (More than 60 years old, HR: 0.70; 95% CI: 0.61–0.81), patients with physical disability or brain lesion disorder (HR: 0.83; 95% CI: 0.71–0.98), had lower risk of turnover to low level. Meanwhile, patients with higher CCI (HR: 1.02; 95% CI: 1.01–1.04), lumbar stenosis (HR: 1.37; 95% CI: 1.27–1.48) and spondylolisthesis (HR: 1.14; 95% CI: 1.00–1.29) had higher risk of turnover to low level. Increase in the number of outpatient visit was significant for higher risk of turnover to low level (HR: 1.003; 95% CI: 1.000–1.006). Besides, entry year and duration of anxiolytics prescription was significant.

In patients having low level of continuity of care at baseline, increase in the number of outpatient visit was significant for higher rates of turnover to high level (HR: 1.05; 95% CI: 1.04–1.06). Besides, entry year, duration of anxiolytics and antipsychotics prescription and the number of usage of epidural steroids injection was significant.

## Discussion

This study investigated whether the continuity of care is associated with lower risk of lumbar surgery in patients with disc herniation more than 6 months. Considering continuity of care and its associated covariates varies over times, continuity of care was treated as time-varying exposures and hospitalization and the number of outpatient visits were treated as time-varying confounders. To estimate the association more accurately, a marginal structural model was used. As a result, the high level of continuity of care was associated with lower risk of lumbar surgery (HR, 0.23; 95% CI, 0.20–0.27). Despite several concerns with musculoskeletal diseases in public health^18,19^, the study of continuity of care in musculoskeletal diseases is rare. To our best of knowledge, this is the first cohort study investigating the effects of continuity of care on a musculoskeletal disease. Furthermore, in terms of methodology, this is the first study using a marginal structural model in the field of continuity of care.

Although there are no studies on the effects continuity of care on disc herniation, the results from this study are consistent with previous studies in the view of management of chronic non-communicable diseases. In the management of lumbar disc herniation, patient's behaviour, such as exercise^20^ and avoidance of fear^21,22^ is associated with disease outcomes. The continuity of care is associated with change in patient's behaviour, such as exercise^23^ and adherence to medications^24^. This could be explained by the relationship between patients and their healthcare providers, which is supported by interpersonal continuity of care^8^. With these characteristics of continuity of care, better quality of management might be provided.

Meanwhile, the association was tremendous compared to previous studies on continuity of care. For example, the association between COC and all-cause mortality in Korean patients with hypertension, diabetes, hypercholesterolemia, or their complications was 0.89 in HR^25^. In COPD patients, the HR was 0.81^26^. This may be due to the nature of the disease and outcome. For disc herniation, conservative usual care alone can reduce pain and improve functionality by half in a year^4^. For usual care, patient’s participation, such as the adherence to treatment is important^27,28^. Thus, if COC had a strong effect on such aspects, it may have significantly mediated the effectiveness of usual care. This should be addressed in the further study. Otherwise, it may be due to the calculation of continuity of care index. For example, a patient with severe symptoms might have complex needs^29^. As a result, the patient may have visited various types of providers and medical institution. Accordingly, we performed sensitivity analysis calculating COC only with outpatient visits to primary care and disc herniation related specialty and the HR increases to 0.50. This demonstrates the importance of identifying complex needs according to disease in COC studies.

We considered the continuity of care has time-varying characteristics in the real-world. Van Walraven et al.^13^ reviewed the bias that occur when these characteristics are not considered in the COC study. Furthermore, we considered that past and present continuity of care is associated by a variety of factors. To estimate the causal estimate of continuity of care in the observational studies, we used marginal structural model with time-dependent analysis and prove the assumption with exploratory analysis and causal mediation analysis. Continuity of care steadily decrease over time. Moreover, patients having high level of continuity of care at baseline are more likely to turn over. This means it is relatively difficult to maintain high levels of continuity of care. Especially, Van Walraven, et al.^13^ pointed out that the more severe the disease is, the lower continuity of care can be, which means the reverse causality. In this study, lumbar stenosis, spondylolisthesis and hospitalization was associated with turnover from high to low level of continuity of care.

On the other hand, some results were rather contradicted to assumption. Older patients and patients with disability had lower rates of turnover to low level. This suggested age, disability and comorbidity (i.e., lumbar stenosis and spondylolisthesis) can act differently for the changes in continuity of care. Furthermore, as the number of outpatient visits increases, patients are likely to turnover from high level to low level, and vice versa. This means that the number of outpatient visits worked differently for each level of continuity of care. Previous studies assumed the number of outpatient visits as proxy of severity^11,15,30^. But this result contradicts such assumption. But this is as far as an exploratory analysis and interpreting coefficients of all variables in the model might be a biased approach^31^. These findings should be addressed in further studies.

We performed marginal structural model and multivariate adjusted model both, however, there was little difference in the estimates. This might be due to the following reasons. The impact of time-varying confounder might be too weak. Although the indirect effect of previous continuity of care to subsequent continuity of care was significant, the proportion mediated was less than 0.03 percent. Also, the distribution of IPTW was so narrow. The 1st and 3rd quartile of IPTW were 0.9759 and 1.001. In this respect, unmeasured confounders should be addressed in the further research. Only hospitalization and the number of outpatient visits were adjusted as time-varying confounders. However, the continuity of care consists of comprehensive concepts, including behaviour and psychological factors. These factors might change with time, and it interacts with continuity of care. Furthermore, both pain and functional limitations should be considered in adjusting the comorbidity of disc herniation. However, due to the limitation of data, these confounders could not be directly measured in patients.

There is another limitation to consider. There might be a problem with validity of the diagnosis code. Magnetic resonance imaging (MRI) is recommended as most appropriate tests to confirm the presence of lumbar disc herniation^1^. However, MRI was not covered by insurance for lumbar disc herniation during study periods. Thus, MRI usage records were not available in this study. To address this problem, sensitivity analysis was performed for the patients who visited hospital at least once within 6 months and the results were consistent. However, this approach does not completely solve the problem.

## Conclusion

High level of continuity of care is associated with a lower risk of lumbar surgery. It is important to consider time-varying characteristics of continuity of care and covariates in a study on continuity of care.

## Methods

### Data source

The National Health Insurance Service–National Sample Cohort (NHIS-NSC) 2.0 DB was used for this study. The samples of NHIS-NSC were randomly extracted from the Korean population using national health insurance or medical aids, based on 2,142 levels of stratification by sex, age, income, and region. One million participants were extracted, comprising 2% of the total eligible Korean population, followed from 2002 to 2015. National health insurance in Korea is a single-payer system; as a result, this cohort represents the whole population in Korea. The cohort data comprised: (i) participant’s demographics, (ii) medical information such as medical procedure, prescription, and diagnostic codes using the International Classification of Disease-10 (ICD-10), (iii) health examination results, and (iv) medical institution information^32^.

### Study population

The population of this study includes patients with moderate disc herniation who need long-term management. Patients included in the study were (i) Patients first diagnosed with Lumbar disc herniation (M51, main diagnosis) from January 01, 2004 to June 31, 2013. The usage of M51 for diagnostic codes for Lumbar disc herniation was suggested by Health Insurance Review & Assessment Service in Korea^33^. (ii) Patients under 20 years old at the onset were excluded, because their behaviour might be quite different from other ages^34^.

To define moderate severity, we included patients (iii) whose episode was longer than 6 months and (iv) did not undergo surgery within 6 months. For patients with severe symptoms, surgery before 6 months is recommended^1^. Therefore, if patients had surgery records within 6 months from onset, this could be a sign of severe conditions. (v) Patients who were pregnant and those with red flag signs (Supplementary Table S1) within six months were excluded, because patients with these symptoms are considered emergency situations, or the resulting surgery may not be preventable with management which is of interest in this study. After six months, these conditions were considered as censoring. (vi) Furthermore, patients who had more than four outpatient visits within 6 months were included, because the number of visits could be an indication of severity^11,15,30^ and when the number of outpatient visits is less than four, the calculation of continuity of care became unstable^35^.

### Continuity of care

To measure continuity of care, Bice-Boxerman Continuity of Care index^36^ was used. It is continuous index ranging from zero to one. When index is closer to one, it means the patient had more continuity of care. This index reflects total outpatient visits, concentration, and variance of outpatient visits to health care provider (Supplementary Equation and Supplementary Table S5). It is widely used for measuring continuity of care^8^. To calculate continuity of care index, the records of outpatient visits before surgery were used. Outpatient visits to all types of physicians were used for calculating index in the main analysis. Restricting types of outpatients visits for index calculation was performed in sensitivity analysis.

The time interval for calculating index was set to three months, considering that this interval was used for the primary^3,37^ and secondary endpoint^2^ of lumbar disc herniation study. To our best of knowledge, there is no commonly used criteria for differentiating high and low levels of continuity of care. Several studies used various criteria according to their study design, such as arbitrary^38^, and relative cut-point (e.g. median^25^ or quartile^39^). To set the criteria, the goodness-of-fit between arbitrary and relative cut-point was compared using concordance index. For arbitrary cut-point, 0.5 was used. For relative cut-point, the median of continuity of care index in each time interval (3 months) was calculated. As a result, a model with the median cut-point had better goodness-of-fit than 0.5 cut-point (concordance index, median cut-point: 0.75; 0.5 cut-point: 0.71) was used in the main analysis. The analysis with 0.5 cut-point was conducted as sensitivity analysis.

### Outcome

Spinal fusion (N0466, N1466, N0469, N1460, N1469 and N2470), open discectomy (N1493), percutaneous endoscopic lumbar discectomy (PELD; N1494), and Laminectomy (N1499 and N2499) were included^5^.

### Covariates

Demographic characteristics, such as sex (female and male), age, residence (metropolitan, urban and rural), income (high, middle and low), and current working status (yes or no) were included. To minimize residual confounders, age was included as both a continuous variable and a categorical variable. Income was included from basic livelihood (0th) to 3rd as low, 4th to 7th as middle, and 8th to 10th as high. Current working status was defined from insurance eligibility status.

For comorbidity adjustment, Charlson comorbidity index (CCI) is most widely used. However, a musculoskeletal disease is not included in calculation of CCI score. So, in addition to including CCI as a continuous variable, we included past history of rheumatoid arthritis (M05 and M06), osteoarthritis (From M15 to M19), spondylolisthesis (M431), spinal stenosis (M480), and osteoporosis (M80, M81 and M82). Moreover, total prescription days of non-steroidal anti-inflammatory drugs, glucocorticosteroids, opioids, antiepileptics, antidepressants, anxiolytics, hypnotics, sedatives and antipsychotics and the number of epidural spinal injection usage (Supplementary Table S7) from onset to baseline were included. For psycholeptic drugs and antidepressants, a patient can be prescribed with other diseases as main diagnosis. Thus, all prescription records were used for these drugs.

The number of outpatient visits and hospitalization with lumbar disc herniation as main diagnosis during the follow-up period was considered as time-varying exposure. Continuity of care reduces the risk of hospitalization^12^, even in musculoskeletal disease^39^. Furthermore, we considered that after hospitalization, the pattern of outpatient visit could change. For the number of outpatient visits, several studies on continuity of care included it as a proxy of severity^11,15,30^. Also, we considered that continuity of care could affect the resource uses. The number of outpatient visits and hospitalization were included in the model cumulatively.

### Statistical analysis

**Figure 4:**
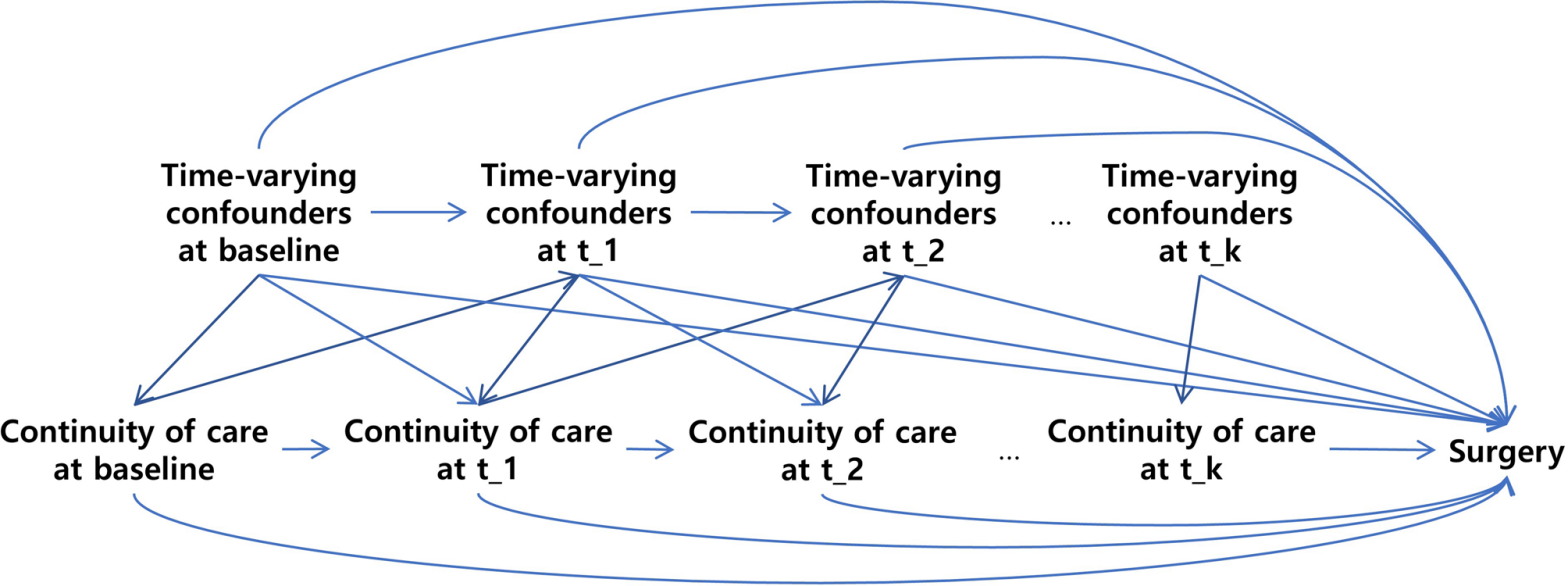
Directed acyclic graph of the association between continuity of care and lumbar surgery while hospitalization acting as time-varying confounder. The causal diagram is presented with directed acyclic graph. Continuity of care is time-varying exposure. Time-varying confounders (i.e., hospitalization and the number of outpatient visits) is associated with previous continuity of care and confounds the association between subsequent continuity of care and Surgery. Outcome is the event of the lumbar surgery.

The causal diagram of the study is presented in Fig. 4. This represents association between continuity of care, time-varying confounders and outcome. Continuity of care changes with time and previous level of continuity of care (e.g., time at t_k-1) affect the subsequent time-varying confounder (e.g., time at t_k). The affected time-varying confounder confounds the association between current level of continuity of care (e.g., time at t_k) and surgery. We tested whether the number of outpatient visits and hospitalization meets the assumptions for time-varying confounders. In short, we use linear mixed model with the number of outpatient visits as outcome and survival analysis with the event of hospitalization as outcome. Subsequently, we used causal mediation analysis^40^ to test whether time-varying confounder mediates the association between previous and subsequent level of continuity of care (Detailed explanation is in Supplementary Methods, Supplementary Fig. S3 and Supplementary Table S7).

After confirming that assumptions were met, marginal structural model with time dependent survival analysis was used. Censoring criteria was one of the following: (i) death, (ii) pregnancy and red flag signs (Supplementary Table S1) and (iii) end of the follow-up (December 31, 2015). The follow-up starts six months from onset. Because the episode more than six months was considered as appropriate for a criteria of moderate severity and surgery within six months might be for severe patients, preventing it was not the focus of this study^1^. The population was observed daily until the events or censoring.

To perform marginal structural model, stabilized inverse probability of treatment weighting (IPTW) was estimated with time-varying confounders as the denominator. After estimation, the time dependent survival analysis, weighted by IPTW, was performed. The result of the marginal structural model was compared with the multivariate adjusted (unweighted) model. In adjusting baseline covariates, the proportional hazards assumption for baseline covariates was tested by log–log survival curve and Schoenfeld residuals. If any assumption failed, interaction with time was included.

Subgroup analysis was performed according to sex and age (20–39, 40–59 or more than 60 years old). Considering that the concept of continuity of care is associated with behavior of patients, which might be influenced by socioeconomic factors, it was stratified by income (high, middle or low) and residence (metropolitan or urban and rural). For comorbidity, the prevalence of stenosis (yes or no), which is considered to be most important factor for prognosis, was used.

For exploratory purposes, (i) the rates of changes to other levels of continuity of care from levels of continuity of care at baseline was investigated. The first turnover to the other level of continuity of care was considered as an event. For example, in patients having low level of continuity of care at baseline, the first turn over to high level of continuity of care during follow up was considered as an event. Cumulative incidence curve was plotted, and the HR was estimated by Cox proportional hazard model. (ii) Next, which factors might influence the probability of changes in levels of continuity of care was investigated. The population was split by levels of continuity of care at baseline. Cox proportional hazard model was used. Covariates at baseline were included in model. Prescription duration was included as weekly basis. If the proportional hazards assumption failed, interaction term with follow-up time was adjusted additionally.

Sensitivity analysis was performed for the continuity of care index and validity of diagnosis. (i) First, analysis with 0.5 cut-point in differentiating high and low levels of continuity of care was performed. The purpose of this analysis is to confirm which of 0.5 or medium is more suitable criteria for differentiating the high and low level. The goodness of fit using concordance index was compared with medium criteria. This analysis was performed only for the main analysis set. (ii) Second, if a patient had complex needs, the patient would likely to visit multiple medical specialties, especially for patients with severe symptoms. This could easily conflate the index and inflated the association. Thus, index was calculated only with outpatient visit to primary care. (iii) Third, index was calculated only with outpatient visit to primary care with related specialties on disc herniation (General practitioner, internal medicine, neurology, psychiatry, surgery, orthopedic surgery, neurosurgery, anesthesiology, radiology, rehabilitation medicine, family medicine, emergency medicine, occupational and environmental medicine or Korean traditional medicine). To calculate index accurately, the analysis on each index was restricted to patients who had more than 4 visits used for calculation within 6 months from onset. For example, if index was calculated only with outpatient visits to primary care, patients who visited primary care more than 4 times were analyzed. The cumulative number of outpatient visit was included in the model as covariates according to each types of index.

For the validity of diagnosis, (i) magnetic resonance imaging (MRI) is recommended as most appropriate tests to confirm the presence of lumbar disc herniation^1^. However, MRI was not covered by insurance for lumbar disc herniation during study periods. Thus, MRI usage records were not available in this study. Considering about 83% of MRI equipment in Korea are distributed in the hospital^41^, the analysis was restricted to the patients who visited hospital at least once within 6 months (n = 8544). (ii) Second, change in diagnostic codes system for claims in Korea was considered. Before 2010, standardized diagnostic codes for dual-licensed system were not implemented. In Korea, doctors of Western Medicine and doctors of Traditional Korean Medicine separately diagnose and treat patients. Before 2010, they used different diagnostic code system for claims. To address this issue, only patients registered in the cohort after 2010 (n = 10,155) were included in the analysis. For each analysis set, the analysis was performed with index calculated by all type of outpatient visits, primary care visits and primary care visits with related specialties.

All p-values were two-sided, and a value less than 0.05 was considered statistically significant. Statistical analyses were performed using SAS version 9.3 (SAS Institute, Cary, NC, USA), and R studio 1.0.136 (© 2009–2016 RStudio, Inc.).

## Supplementary Information

Supplementary Information

## Data Availability

The NHIS-NSC is provided by the National Health Insurance Service in Korea. For patient’s privacy, access to the data is available only for certified researchers in South Korea. The study protocol was approved by the Institutional Review Board of Seoul National University (IRB No. E1810/002-002) and followed relevant guidelines. The requirement for informed consent from the study population was waived by the same IRB.
