# Supplementary material for: The association between continuity of care and surgery in lumbar disc herniation patients
Source: Sci Rep. 2021 Mar 10;11:5550. doi: 10.1038/s41598-021-85064-1 (PMC7946938; doi:10.1038/s41598-021-85064-1)
Supplement: Supplementary file 1 — Supplementary Information [file 41598_2021_85064_MOESM1_ESM.docx]

**The association between continuity of care and surgery in lumbar disc herniation patients.**

Eun-San Kim^1^, Chang-yup Kim^1^*

^1^ Graduate School of Public Health, Seoul National University, 1 Gwanak-ro, Gwanak-gu, Seoul 08826, Republic of Korea

Corresponding author:

Chang-yup Kim

Graduate School of Public Health, Seoul National University, 1 Gwanak-ro, Gwanak-gu, Seoul 08826, Republic of Korea.

E-mail: [cykim@snu.ac.kr](mailto:cykim@snu.ac.kr)

**Supplementary Table S1. The number of patients in cohort by follow-up period.**

|  | **Follow-up year** | | | | | | | | | | | |
| --- | --- | --- | --- | --- | --- | --- | --- | --- | --- | --- | --- | --- |
|  | **0 - 1** | **1 - 2** | **2 - 3** | **3 - 4** | **4 - 5** | **5 - 6** | **6 - 7** | **7 - 8** | **8 - 9** | **9 - 10** | **10 - 11** | **11 - 12** |
| **Total** | 29,061 | 25,030 | 22,165 | 18,420 | 15,080 | 11,998 | 9,434 | 7,241 | 5,385 | 3,673 | 2,013 | 619 |
| **High level of continuity of care** | 19,613 | 14,507 | 11,231 | 9,231 | 7,636 | 6,059 | 4,743 | 3,689 | 2,700 | 1,836 | 1,015 | 313 |
| **Low level of continuity of care** | 9,448 | 10,523 | 10,934 | 9,189 | 7,444 | 5,939 | 4,691 | 3,552 | 2,685 | 1,837 | 998 | 306 |
| This table shows the number of patients in cohort as the follow-up years passed. The criterion of high and low level of continuity of care is medium of continuity of care index | | | | | | | | | | | | |

**Supplementary Figure S1. The distribution of IPTW from the start of cohort**


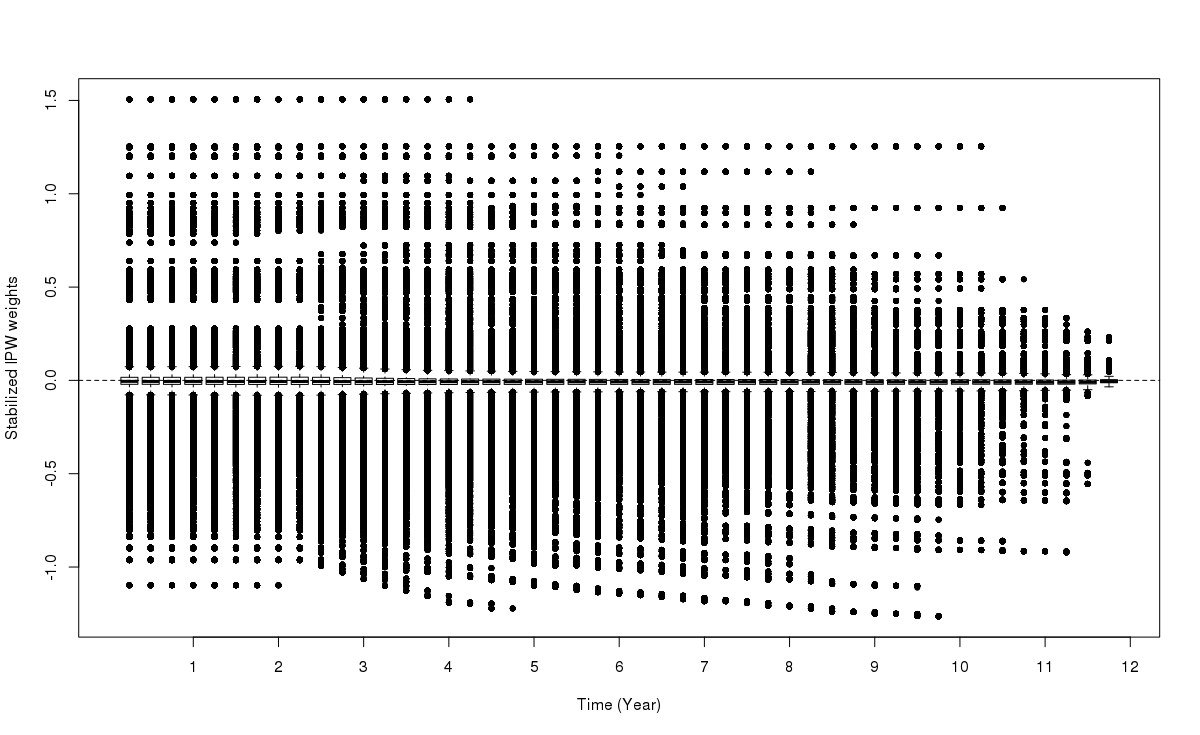


The distribution of IPTW during periods is presented with box plot. IPTW is summarized by 3 months interval. The top and bottom line of the box plot represents 25^th^ percentile and 75^th^ percentile each. The dots represent outliers.

**Supplementary Table S2. Descriptive characteristics of the IPTW**

| Minimum | 1^st^ quartile | Median | Mean | 3^rd^ quartile | Maximum |
| --- | --- | --- | --- | --- | --- |
| 0.282 | 0.979 | 0.993 | 0.999 | 1.001 | 4.501 |

**Supplementary Figure S2. Unadjusted cumulative incidence curve of turnover to other levels of continuity of care from baseline level of continuity of care.**


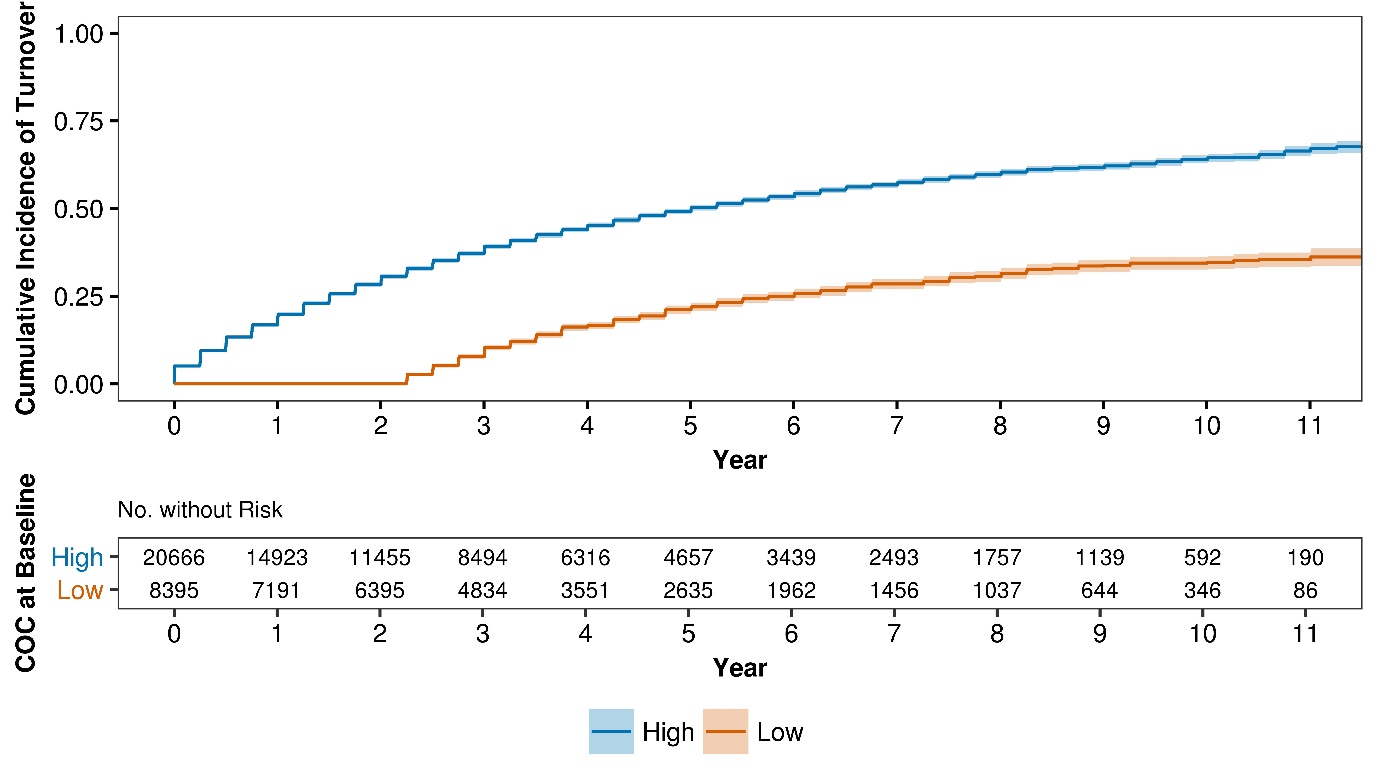


The unadjusted cumulative incidence curve of turnover to other levels of continuity of care from baseline level of continuity of care was plotted. The first turnover to the other level of continuity of care was considered as event. Patients having high level of continuity of care at baseline is presented with blue line and low level of continuity of care is presented with red line. The curve is presented with 95% confidence interval.

**Supplementary Table S3. Analysis with turnover to other level of continuity of care by continuity of care level at baseline**

|  |  | High level at baseline | High level at baseline |
| --- | --- | --- | --- |
| Sex | Female | 1.04 (0.97 - 1.11) | 0.95 (0.69 - 1.30) |
| Age | 20-29 | reference | reference |
|  | 30-39 | 0.88 (0.77 - 1.02) | 0.73 (0.42 - 1.27) |
|  | 40-49 | 0.82 (0.72 – 0.94) | 1.04 (0.60 - 1.79) |
|  | 50-59 | 0.78 (0.68 – 0.89) | 0.99 (0.56 - 1.75) |
|  | 60- | 0.70 (0.61 – 0.81) | 1.13 (0.61 – 2.09) |
| Residence | Metropolitan | Reference | reference |
|  | Urban | 0.96 (0.90 - 1.02) | 0.93 (0.69 - 1.25) |
|  | Rural | 1.02 (0.92 - 1.12) | 0.86 (0.54 - 1.37) |
| Income | High | Reference | reference |
|  | Middle | 1.02 (0.95 - 1.09) | 1.05 (0.76 - 1.47) |
|  | Low | 1.02 (0.95 - 1.10) | 1.07 (0.74 - 1.54) |
| Working status | Working | 0.99 (0.93 - 1.05) | 0.95 (0.70 - 1.27) |
| Entry year | 2004 | Reference | Reference |
|  | 2005 | 0.94 (0.82 - 1.08) | 1.77 (0.99 - 3.18) |
|  | 2006 | 1.01 (0.88 - 1.16) | 1.86 (1.02 - 3.37) |
|  | 2007 | 1.03 (0.89 - 1.18) | 1.49 (0.80 - 2.75) |
|  | 2008 | 1.04 (0.89 - 1.20) | 2.15 (1.07 – 4.32) |
|  | 2009 | 1.05 (0.90 - 1.22) | 2.69 (1.28 – 5.66) |
|  | 2010 | 1.10 (0.94 - 1.27) | 2.75 (1.17 – 6.50) |
|  | 2011 | 1.23 (1.05 – 1.43) | 4.30 (1.41 – 13.10) |
|  | 2012 | 1.32 (1.12 – 1.56) | 1.92 (0.34 – 10.92) |
|  | 2013 | 1.69 (1.42 – 2.01) | 1.80 (0.01 – 613.94) |
| Disability | Yes | 0.83 (0.71 – 0.98) | 0.85 (0.37 - 1.96) |
| Comorbidity | CCI | 1.02 (1.01 – 1.04) | 1.01 (0.97 - 1.05) |
|  | Osteoarthritis | 1.05 (0.98 - 1.12) | 0.92 (0.66 - 1.28) |
|  | Rheumatoid | 0.99 (0.91 - 1.08) | 0.94 (0.59 - 1.52) |
|  | Osteoporosis | 0.96 (0.89 - 1.05) | 1.30 (0.85 – 2.01) |
|  | Lumbar stenosis | 1.37 (1.27 – 1.48) | 0.87 (0.62 - 1.24) |
|  | Spondylolisthesis | 1.14 (1.00 – 1.29) | 0.89 (0.47 - 1.69) |
| Number of usage | Outpatient visit | 1.003 (1.000 – 1.006) | 1.05 (1.04 – 1.06) |
|  | Hospitalization | 1.13 (1.04 - 1.22) | 0.93 (0.82 – 1.04) |
|  | Epidural steroid injection | 1.00 (0.99 - 1.01) | 0.96 (0.93 – 0.99) |
| Prescribed weeks | Non-steroidal anti-inflammatory drugs | 1.00 (0.99 – 1.00) | 0.99 (0.96 – 1.02) |
|  | Glucocorticosteroids | 1.02 (0.99 - 1.05) | 0.92 (0.82 - 1.03) |
|  | Opioids | 0.99 (0.98 – 1.01) | 1.01 (0.99 – 1.03) |
|  | Anticonvulsants | 0.99 (0.98 – 1.01) | 1.00 (0.97 – 1.03) |
|  | Antidepressants | 1.00 (0.99 – 1.00) | 1.00 (0.99 – 1.01) |
|  | Anxiolytics | 1.004 (1.001 – 1.007) | 1.008 (1.000 - 1.016) |
|  | Hypnotics and sedatives | 1.01 (1.00 – 1.02) | 0.99 (0.96 – 1.01) |
|  | Antipsychotics | 1.00 (0.99 – 1.00) | 1.020 (1.002 – 1.038) |
| The cohort was split by level of continuity of care at baseline. In each cohort, the first turnover to the other level of continuity of care was defined as event (i.e., The event in the patients with high level at baseline: Turnover from high level to low level; The event in the patients with low level at baseline: Turnover from low level to high level). Cox proportional hazard model was used. Covariates at baseline were included in model. Prescription duration was included as weekly basis. All association is presented as hazard ratio (HR) and 95% confidence interval (CI). | | | |

**Supplementary Table S4. Diagnosis as exclusion criteria**

| ICD-10 | Disease |
| --- | --- |
| C00-D48 | Neoplasms |
| G061 | Intraspinal abscess and granuloma |
| M45 | Ankylosing spondylitis |
| M86 | Osteomyelitis |
| M46.0 | Spinal enthesopathy |
| M46.9 | Inflammatory spondylopathy, unspecified |
| M89.6 | Osteopathy after poliomyelitis |
| M90.2 | Osteopathy in other infectious diseases classified elsewhere |
| O00-O99 | Pregnancy, childbirth and the puerperium |
| S32 | Fracture of lumbar spine and pelvis |
| S33.0 | Traumatic rupture of lumbar intervertebral disc |
| S34.1 | Other injury of lumbar spinal cord |
| S34.3 | Injury of cauda equina |
| T08.1 | Fracture of spine, level unspecified, open |
| T09.3 | Injury of spinal cord, level unspecified |
| V01-V09 | Transport accidents |

**Supplementary Equation. The equation of Bice-Boxerman Continuity of Care index**

$$COC=\frac{\sum_{i=1}^{M} n_{i}^{2}-N}{N(N-1)}$$

M: The number of providers; n_i_: The number of visits to provider I; N: Total number of visits during episode.

**Supplementary Table S5. The example of Bice-Boxerman Continuity of Care index for a patient with 6 visits.**

| The number of providers | Providers | | | | | | Index |
| --- | --- | --- | --- | --- | --- | --- | --- |
|  | A | B | C | D | E | F |  |
| 1 | 6 |  |  |  |  |  | 1 |
| 2 | 1 | 5 |  |  |  |  | 0.67 |
| 2 | 2 | 4 |  |  |  |  | 0.47 |
| 2 | 3 | 3 |  |  |  |  | 0.4 |
| 3 | 1 | 1 | 4 |  |  |  | 0.4 |
| 3 | 1 | 2 | 3 |  |  |  | 0.27 |
| 3 | 2 | 2 | 2 |  |  |  | 0.2 |
| 4 | 1 | 1 | 1 | 3 |  |  | 0.2 |
| 4 | 1 | 1 | 2 | 2 |  |  | 0.13 |
| 5 | 1 | 1 | 1 | 1 | 2 |  | 0.07 |
| 6 | 1 | 1 | 1 | 1 | 1 | 1 | 0 |
|  | | | | | | | |

**Supplementary Table S6. ATC code of medication and procedure code of epidural steroid injection**

| Medications | | | |
| --- | --- | --- | --- |
| ATC code | Name | ATC code | Name |
| M01 | Non-Steroidal Anti-Inflammatory Drugs | N06A | Antidepressants |
| H02 | Glucocorticosteroids | N05B | Anxiolytics |
| N02A | Opioids | N05C | Hypnotics and sedatives |
| N03 | Anticonvulsants | N05A | Antipsychotics |
| Epidural steroid injection | | | |
| Code | Name | Code | Name |
| LA253 | Lumbar or Sacral Plexus | LA356 | Gray Rami Communicans |
| LA322 | Lumbar and/or Caudal | LA357 | Posterior Division of Spinal Nerve |
| LA354 | Selective Spinal Nerve Root | LA358 | Posterior Medial Branch |
| LA355 | DRG (Dorsal Root Ganglion) | LA359 | Facet Joint |

In NHIS-NSC, the record of medication is presented as main ingredient code (variable name: gnl_nm_cd). For commensurability, the authors translated it to ATC code.

**Supplementary Methods. Proving the assumptions of time-varying confounder.**

The author investigated whether hospitalization and the number of outpatient visits are time-varying confounder in the causal pathway between continuity of care and surgery. The author tested whether a) continuity of care is associated with time-varying confounders; and b) previous levels of continuity of care (t_k-1) is associated with subsequent levels of continuity of care (t_k) and the time-varying confounders mediates the association (Supplementary Figure S5).

In order to prove the association between continuity of care and the time-varying confounders, the time-dependent survival analysis and linear mixed model were used. In the survival analysis, the outcome was the event of hospitalization. The same dataset with main analysis was used, except that the data after hospitalization were censored. In the linear mixed model, using same dataset with main analysis duplicates outcome. For example, if a patient was observed for a 1 year and using a day time interval makes the number of observations 365. The estimate is not biased. However, this make the confidence interval too narrow and the result get significant even if the effect is quite a small. Furthermore, this requires a lot of resources for computation. Thus, the patient's episode was summarized by 3 months intervals. The mean value of the cumulative number of outpatients visits during each time interval was used as outcome. In linear mixed model, the random intercept model was used.

To prove the association between previous and subsequent levels of continuity care and the mediation effects, the causal mediation analysis was used. The lagged continuity of care was used as independent variable. The event of hospitalization and the mean value of the cumulative number of outpatients visits during 3 months interval were considered as mediators. All models were adjusted by same confounders with main analysis, except that hospitalization and the number of outpatient visits before baseline were included in the model as time-invariant confounders.

The results are presented in Supplementary Table S7. High level of continuity of care is associated with lower risk of hospitalization (HR: 0.36; 95% CI: 0.33 - 0.39) and lower number of outpatient visits (-4.53; 95% CI: -4.68 – -4.38). Previous levels of continuity of care is associated with subsequent levels of continuity care and the hospitalization (Proportion mediated: 0.02; 95% CI: 0.01 – 0.03) and the number of visits (Proportion mediated: -0.03; 95% CI: -0.03 – -0.02) mediates the effects.

The assumptions for time-varying confounders are met. However, there are important things to consider. This analysis had several limitations for estimating causal effect. First, in the mediation model, the exposure and confounders before t_k-1 also can influence the results. However, only continuity of care at t_k-1 was lagged. Second, there might be lot of unmeasured confounders left. This cohort and model were set up for investigating the association between continuity of care and surgery. As a result, using same cohort and confounders with main analysis had limitation for estimating association between previous and subsequent level of continuity of care.

**Supplementary Figure S3. The Association between time-varying exposure, confounders and outcome**

**
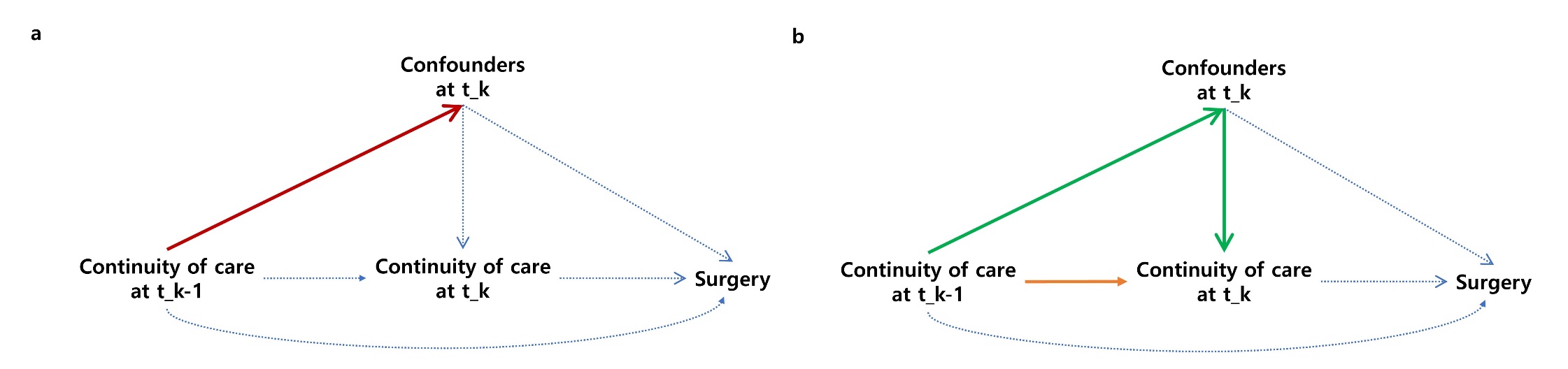
**

The causal diagram is presented with directed acyclic graph. Continuity of care is time-varying exposure. Hospitalization and the number of outpatient visits are time-varying confounder which means that they are associated with previous continuity of care and confounds the association between subsequent continuity of care and Surgery. Outcome is the event of the lumbar surgery. There were two hypotheses for testing the assumptions of time-varying confounders. a) Continuity of care is associated with time-varying confounders; and b) previous levels of continuity of care (t_k-1) is associated with subsequent levels of continuity of care (t_k) and the time-varying confounders mediates the association. The red line is the primary effect, the green line is the mediated effect and the orange line is the direct effect of continuity of care.

**Supplementary Table S7. The association between time-varying exposure, confounders and outcome**

|  | Assumption 1 | Assumption 2^§^ | | | |
| --- | --- | --- | --- | --- | --- |
|  |  | NDE | NIE | TE | Proportion mediated |
| Hospitalization | 0.36 (0.33 – 0.39)^†^ | 95.40 (95.31 – 95.48) | 0.02 (0.01 – 0.03) | 95.41 (95.33 – 95.49) | 0.02 (0.01 – 0.03) |
| Outpatient visits | -4.53 (-4.68 – -4.38)^‡^ | 95.44 (95.36 – 95.51) | -0.03 (-0.03 – -0.02) | 95.41 (95.33 – 95.49) | -0.03 (-0.03 – -0.02) |
| Assumption 1: The continuity of care is associated with time-varying confounders;  Assumption 2: The association between previous and subsequent level of continuity of care is mediated by time-varying confounders;  † The time-dependent survival analysis was used with event of hospitalization as outcome. The result is presented as hazard ratio and 95% confidence interval (CI).  ‡ The linear-mixed model was used with the number of outpatient visits as outcome. The result is presented as marginal change in the number of outpatient visits with 95% CI.  § The causal mediation analysis was used. The natural direct effect (NDE), natural indirect effect (NIE) and total effect (NE) are presented as marginal change in probability of being high levels of continuity of care with 95% CI. The proportion mediated represents the proportion of NIE to TE. All estimates were multiplied with 100 to represent percentage.  Abbreviations, NDE: Natural Direct Effect; NIE: Natural Indirect Effect; TE: Total Effect; | | | | | |
